# Identification of key genes and immune infiltration based on weighted gene co-expression network analysis in vestibular schwannoma

**DOI:** 10.1097/MD.0000000000033470

**Published:** 2022-04-07

**Authors:** Yanpeng Fu, Yaqiong Zhu, Liqing Guo, Yuehui Liu

**Affiliations:** a Department of Otorhinolaryngology Head and Neck Surgery, Second Affiliated Hospital of Nanchang University, Nanchang, China.

**Keywords:** hub genes, immune infiltration, vestibular schwannomas, weighted gene coexpression network analysis

## Abstract

Vestibular schwannomas are the most common tumors of the cerebellopontine angle, but their pathogenesis is still unclear. This study aimed to explore the molecular mechanisms and potential therapeutic target biomarkers in vestibular schwannoma. Two datasets (GSE141801 and GSE54934) were downloaded from the Gene Expression Omnibus database. Weighted gene coexpression network analysis was performed to find the key modules associated with vestibular schwannoma (VS). Functional enrichment analysis was applied to evaluate the gene enrichment signaling pathway in key modules. Protein-protein interaction networks in key modules were constructed using the STRING website. Hub genes were identified by intersecting candidate hub genes in protein-protein interaction network and candidate hub genes in key modules. Single-sample gene set enrichment analysis was utilized to quantify the abundance of tumor-infiltrating immune cells in VSs and normal control nerves. A Random forest classifier was developed based on hub genes identified in this study and validated on an independent dataset (GSE108524). Results of immune cell infiltration were also validated on GSE108524 by gene set enrichment analysis. Eight genes from coexpression modules were identified as hub genes, that is, CCND1, CAV1, GLI1, SOX9, LY86, TLR3, TREM2, and C3AR1, which might be potential therapeutic targets for VS. We also found that there were distinct differences in the infiltration levels of immune cells between VSs and normal control nerves. Overall, our findings may be useful for investigating the mechanisms underlying VS and provide noteworthy directions for future research.

## 1. Introduction

Vestibular schwannoma (VS), a benign tumor deriving from the vestibular branch of the 8th cranial nerve, accounts for about 8% to 10% of all intracranial tumors and for almost 75% tumors in the cerebellopontine angle.^[1]^ The majority of VSs are sporadic (95%), approximately 5% of cases are part of neurofibromatosis type 2 (NF2). The growth of VS may cause sensorineural hearing loss, tinnitus, facial palsy, deafness, brainstem compression, and eventual mortality.^[2]^ So far, the identification of the NF2 gene in 1993 has been the most important contribution to our understanding of VS pathogenesis,^[3]^ but it is likely that other genes contribute to VS formation because the NF2 gene is intact in a significant proportion of cases.^[4]^ To date, the pathologic mechanisms leading to VS have not been fully elucidated, new therapeutic targets for VS are desperately needed.

In recent years, with the development of bioinformatics, genomic sequencing, gene chip, and data mining have increasingly been used to identify novel and important genes when studying disease mechanisms. Currently, some studies have been conducted on VS gene expression profiles, which can be downloaded from gene expression omnibus (GEO). Traditional microarray analysis is often restricted to differentially expressed genes without accounting for the co-regulation and interactions between genes. However, understanding the intricate relationship among genes is important in discovering potential risk factors. Mounting evidence indicates that immune infiltration is one of the vital features in various tumors and neurodegenerative disorder,^[5]^ but only few such studies on immune infiltration in VS have been performed until now. In the present study, weighted gene coexpression network analysis (WGCNA) was used to integrate gene expression and clinical features effectively to reveal the relevance of genes for different samples to find hub genes, and single-sample gene set enrichment analysis (ssGSEA) was developed to calculate the immune scores of VSs and normal nerve tissues based on their gene expression profiles available from public databases.

## 2. Material and methods

### 2.1. Data collection

Publicly available mRNA expression profiles involved in VS were downloaded from the GEO database (https://www.ncbi.nlm.nih.gov/geo/). First, we researched with “vestibular schwannoma” in GEO database to find the expression dataset of VS. after excluding microRNA microarray data, LncRNA microarray data, methylation microarray data and duplicate datasets, the dataset of GSE141801, GSE54934, and GSE108524 was included, in this study, we only analyzed samples of sporadic vestibular schwannomas. Two array datasets were analyzed as the internal cohort in our study, including GSE141801 and GSE54934. GSE141801 generated on the GPL13677 (Affymetrix Human Genome U219 Array) platform and GSE54934 generated on the GPL6244 [Affymetrix Human Gene 1.0 ST Array (transcript (gene) version)]. The batch effect of 2 datasets was removed by “‘SVA’” package (Figure S1, Supplemental Digital Content, http://links.lww.com/MD/I751),^[6]^ with 67 VSs and 16 normal control nerves subsequently included. GSE108524 with platform GPL17586, included 10 VSs and 4 normal control nerves, are selected as the external verification data set in our study. Demographic information for the 3 datasets included in our study were shown in Table S1, Supplemental Digital Content, http://links.lww.com/MD/I752.

### 2.2. Construction of weighted gene coexpression network

The R package “WGCNA”^[7]^ was used to perform the WGCNA on GSE141801 and GSE54934. We selected the top 60% variant genes (10378 genes) for coexpression analysis.

According to the algorithm, scale independence and average connectivity degree of different modules with different power values (the power value ranging from 1 to 20) were performed to find the soft threshold of module analysis. The proper soft threshold was determined when the degree of independence was 0.85 and the module construction proceeded with the appropriate soft threshold. The minimum number of genes was set as 30. After the genes had been divided into different modules, the module eigengenes was determined by the first principal component of the module expression levels. VS was coded as a binary variable. Module-trait associations were estimated using the spearman correlation coefficients between module eigengenes and VS, which facilitates the identification of expression modules highly correlated to VS. Then, we selected modules that had significant positive or negative correlations with VS as key modules and extracted the genes of these modules to perform subsequent analysis.

### 2.3. Enrichment analysis

To further understand the function of the genes in modules related to VS, the gene ontology (GO) annotation and Kyoto encyclopedia of genes and genomes (KEGG) pathway enrichment analyses of the highly correlated modules were carried out via “clusterProfiler” package of R software.^[8]^ The *P* value was adjusted by the Holm–Bonferroni method. The adjusted *P* value cutoff and the q value cutoff was set at 0.05.

### 2.4. Immune cell infiltration score

The ssGSEA that was performed by “GSEA” package^[9]^ was utilized to quantify the abundance of tumor-infiltrating immune cells in VSs and normal control nerves according to the expression of metagenes that are nonoverlapping sets of genes that are representative for specific immune cell subpopulations and are neither expressed in cancer cell lines nor in normal tissue.^[10]^ Then Wilcoxon rank-sum test was used to analyze the immune scores of VSs and normal control nerves to determine differential immune cell types (*P* value < .05) between the 2 groups, and spearman correlation analysis was used to analyze the correlation between immune cell types and samples.

### 2.5. Construction of protein-protein interaction (PPI) network and identification of hub genes

The genes in the key module were uploaded to the STRING (https://string-db.org/) database to construct the PPI network. The result of STRING analysis was visualized by Cytoscape (v3.8.2) software (https://cytoscape.org/).^[11]^ The degree of each node was calculated by cytoscape software, the degree represents the number of nodes related to each node in PPI network. Due to the large number of genes in the key module of this study, in this study, the genes with a degree >20 were considered as candidate hub genes in PPI network. Then we selected genes with an absolute value of module membership for key modules more than 0.80 and an absolute value of gene significance for VS more than 0.8 was selected as candidate hub genes in key modules. Hub genes were identified by intersecting candidate hub genes in PPI network and candidate hub genes in key modules.

### 2.6. Random forest

The R package “randomForest”^[12]^ was used to train the random forest model. Random forest is a classification method that uses multiple trees to train and predict samples. We constructed a random forest model for VS based on hub genes we identified. Internal cohort was used as training set, whereas external verification data set was used as a validation set. The area under the curve (AUC) and the area under the precision-recall curve (AUPRC) was used to evaluate the predictive accuracy of the model.

## 3. Results

### 3.1. WGCNA to identify modules critical to VS

**Figure 1.**
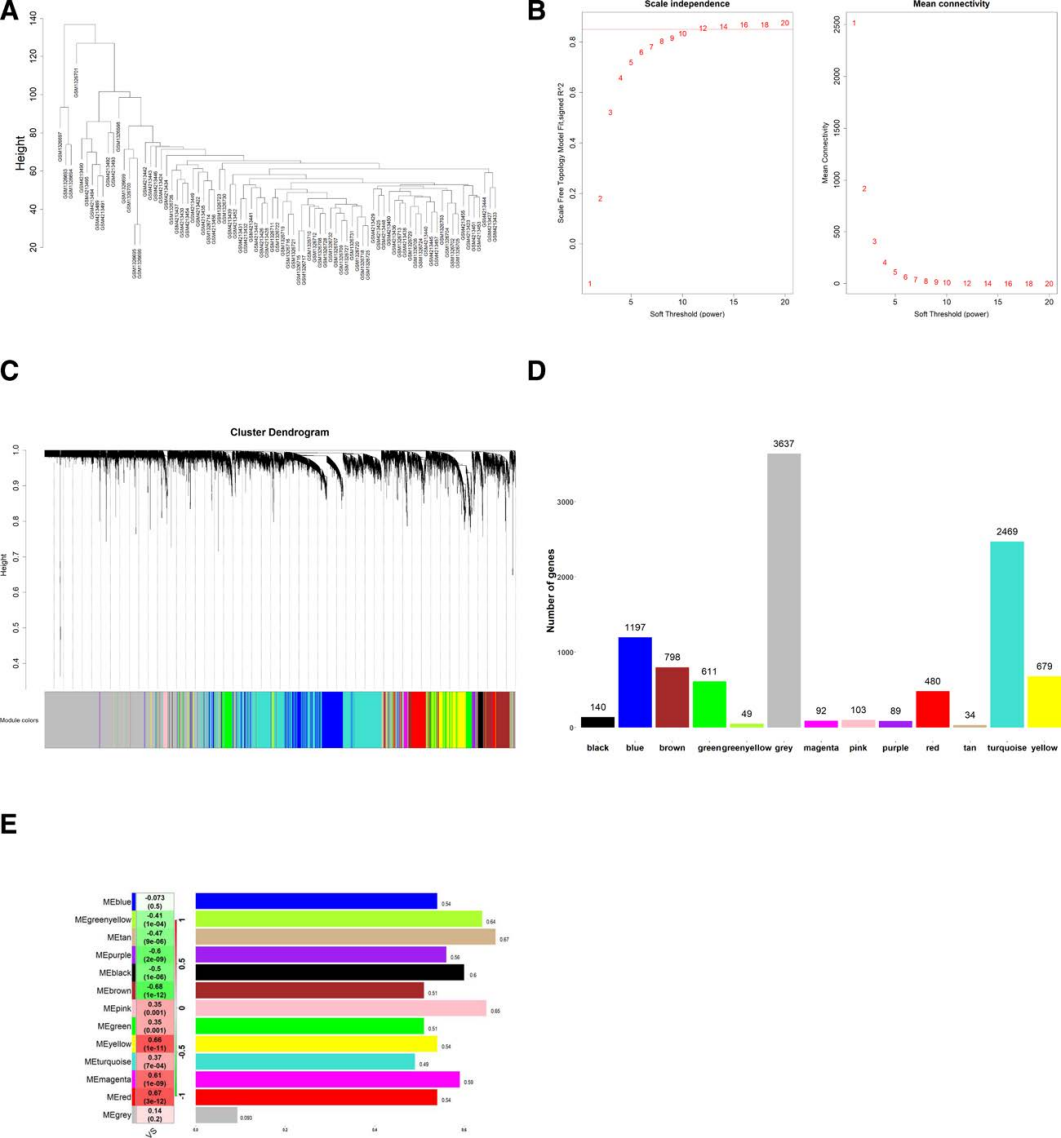
Identification of key modules connected with VS through WGCNA, (A) the clustering dendrograms of samples, all 83 samples were clustered well, (B) determination of the soft-thresholding power (β) in WGCNA, 12 as the final soft threshold for subsequent analysis, (c) construction of genes coexpression modules. Each color represents a module, and each branch represents a module, (D) number of genes in each coexpression module, and (E) Module-trait relationship. Each row corresponds to a module eigengene, and column corresponds to VS. Each cell contains the corresponding correlation and *P* value. VS = vestibular schwannoma, WGCNA = weighted gene coexpression network analysis.

In order to detect the outliers in the internal cohort, a sample hierarchical clustering dendrogram was plotted, as shown in Figure 1A, all 83 samples were clustered well. “pick Soft Threshold” calculation revealed that the suitable soft threshold was 12 (Fig. 1B). Thirteen coexpression modules were identified and are shown in different colors (Fig. 1C and D), all unassigned genes were placed in the gray module.

The correlations between the coexpression modules and clinical traits are shown in Figure 1E. Three modules were most significantly associated with VS. The yellow module (*R* = 0.66, *P* = 1e-11), and red module (*R* = 0.67, *P* = 3e-12) showed a positive correlation, whereas the brown module (r = −0.68, *P* = 1e-12) module showed negative correlations.

### 3.2. Gene function and annotation enrichment analysis of key modules

**Figure 2.**
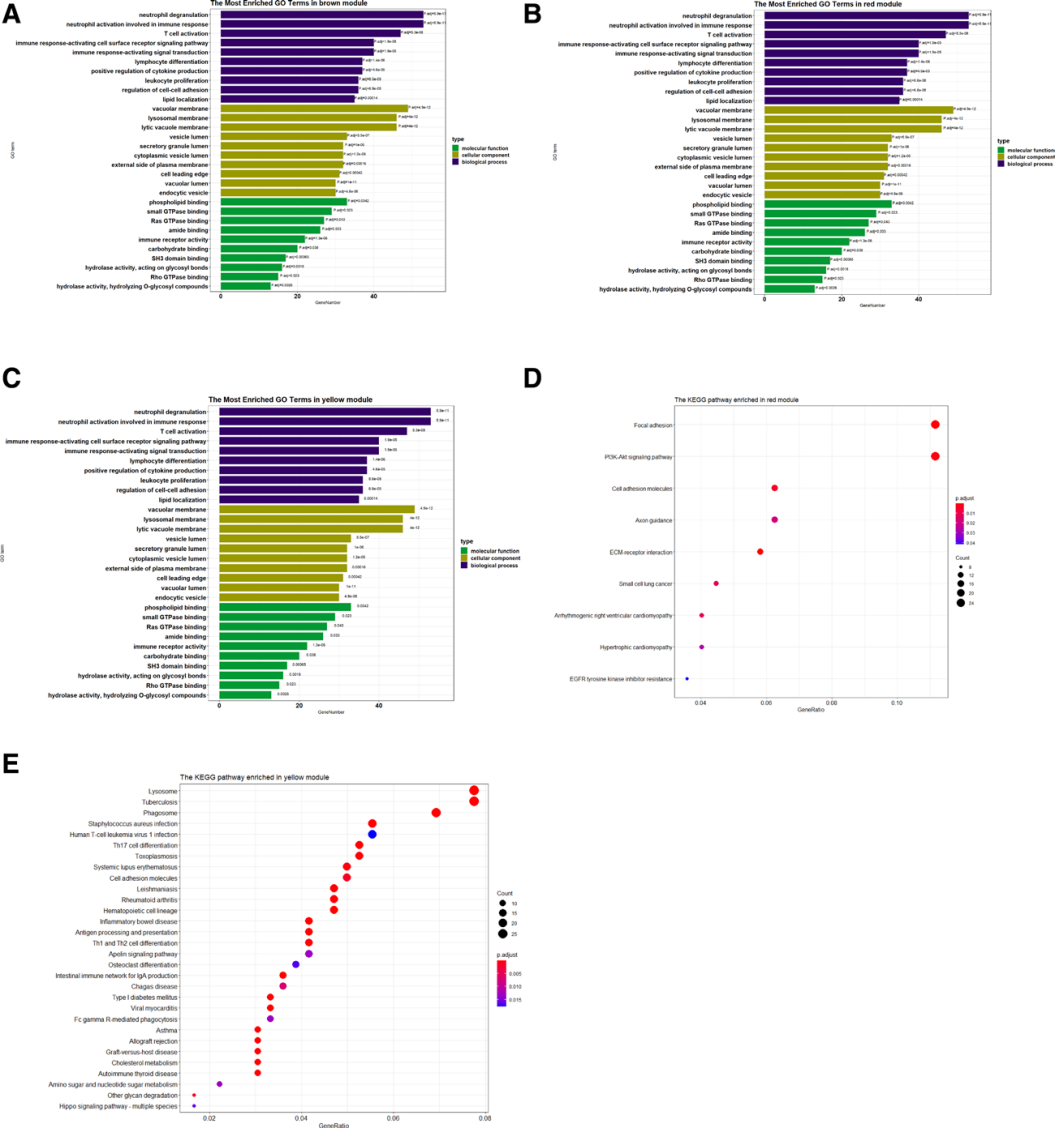
GO enrichment and KEGG analyses for key modules, (A–C) the top 10 terms of GO categories of biological process (BP), cellular component (CC) and molecular function (MF) enriched in brown, red, yellow modules and (D–E) Enrichment analysis of KEGG pathway for red module (D) and yellow module (E), “Gene Ratio” was defined as the ratio of the enriched gene numbers to total gene numbers. GO = gene ontology, KEGG = Kyoto encyclopedia of genes and genomes.

GO term and KEGG pathway enrichment analyses were performed using the “cluster Profiler” package. As shown in Figure 2A–C, GO term enrichment analysis showed that genes in brown module were mainly enriched in collagen-containing extracellular matrix, extracellular matrix organization, extracellular structure organization, axonogenesis, ameboidal-type cell migration, regulation of cell morphogenesis, receptor ligand activity, and signaling receptor activator activity. Genes in red module were mainly enriched in axonogenesis, cell-cell junction, synapse organization, cell junction assembly and positive regulation of neurogenesis. Genes in yellow module were mainly enriched in neutrophil degranulation, neutrophil activation involved in immune response, vacuolar membrane, T cell activation, lysosomal membrane and lytic vacuole membrane.

The KEGG pathway analysis (Fig. 2D–E) showed that genes in red module were mainly enriched in focal adhesion and PI3K-Akt signaling pathway. genes in yellow module were mainly enriched in lysosome, tuberculosis, phagosome, herpes simplex virus 1 infection, MAPK signaling pathway, staphylococcus aureus infection, human T cell leukemia virus 1 infection and salmonella infection. however, no significantly enriched pathways were detected for genes in brown module, which may because genes in brown module were involved in unknown pathway.

### 3.3. Immune cell infiltration

**Figure 3.**
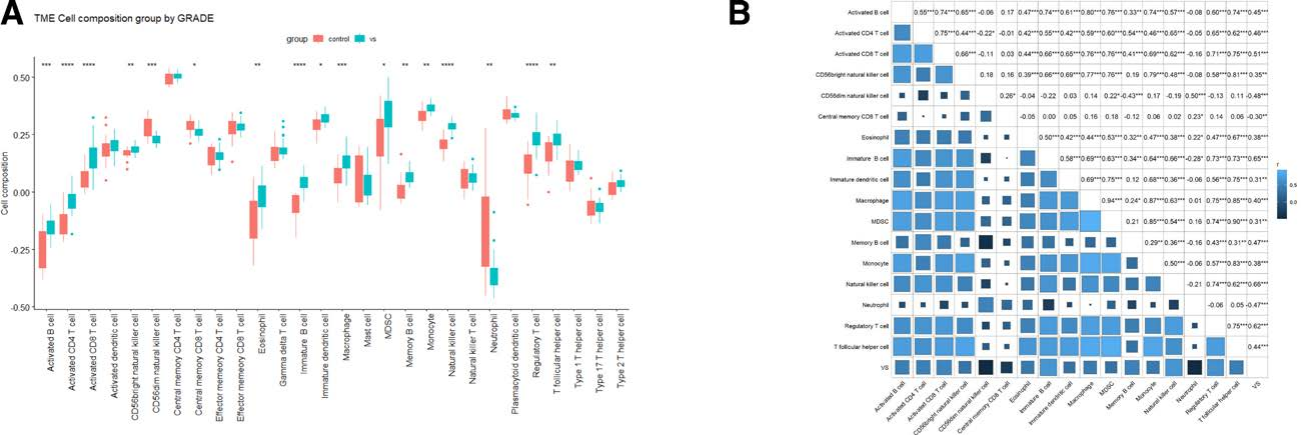
The immune cell infiltration analysis, (A) the landscape of immune cell infiltration based on internal cohort and (B) the correlation of VSs and immune cell types with significant differences between VSs and normal control nerves. (*P < .05, **P < .01, ***P < .001, ****P < .0001). VS = vestibular schwannoma.

We noticed that genes in yellow module were related to inflammation and the immune response. Therefore, we further analyzed the immune cell infiltration landscape of VSs and normal control nerves in internal cohort through ssGSEA (Fig. 3A). Infiltrating levels of 17 kinds of immune cells were significantly different between VSs and normal control nerves. According to the Spearman correlation analysis (Fig. 3B) of these immune cells and disease states, Natural killer cells (*R* = 0.66), immature B cell (*R* = 0.65), and regulatory T cell (*R* = 0.62) were most associated with VS. Together, these results may suggest that the diffidence in immune infiltration between VSs and normal control nerves could contribute to VS development.

### 3.4. PPI network construction and hub genes identification

**Figure 4.**
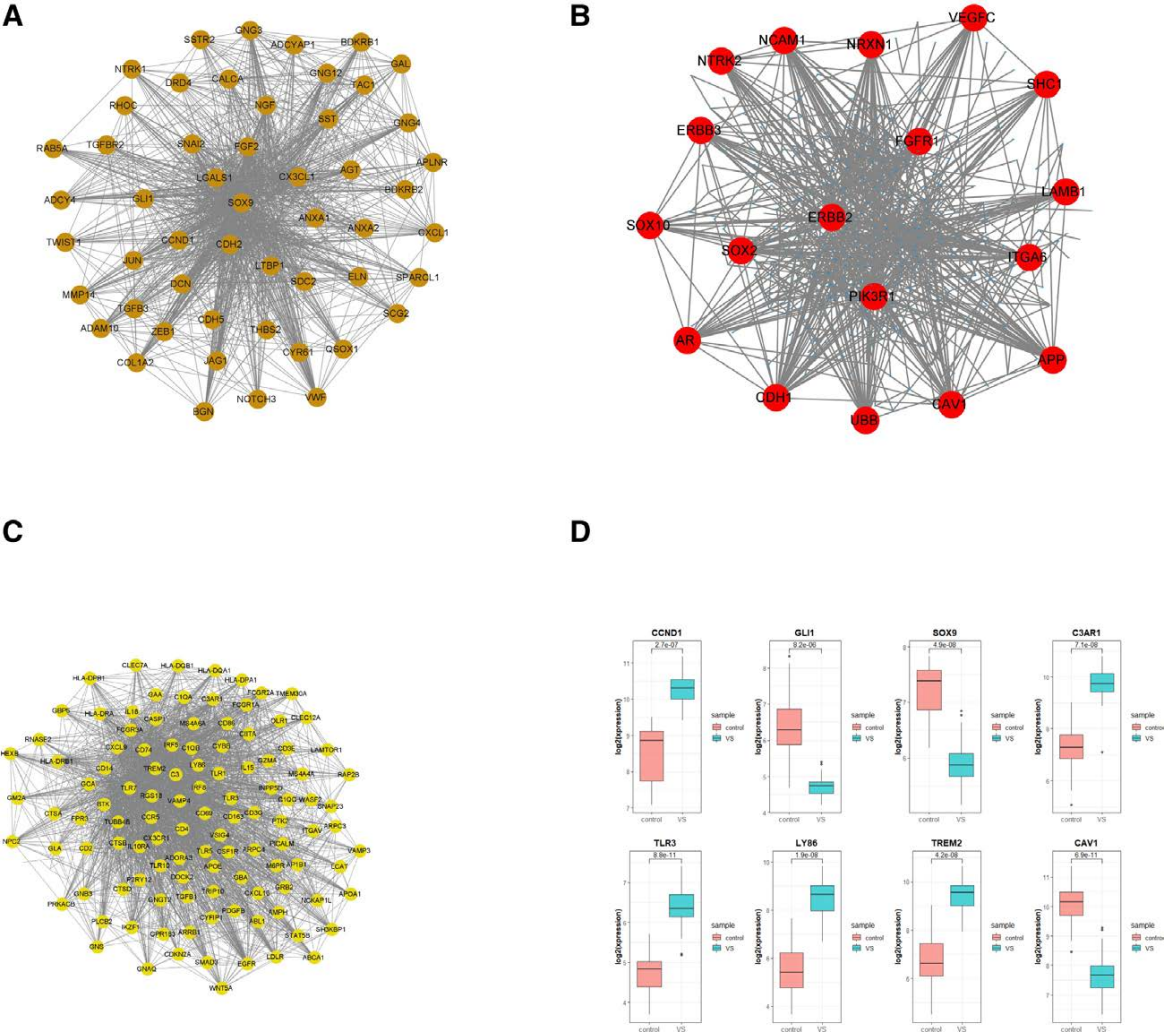
Identification of hub genes, (A–C) genes with degree more than 20 in PPI network in brown, red and yellow modules and (D) Expression level of 8 hub genes in interna dataset. PPI = protein-protein interaction.

The PPI network of genes in the 3 key modules was established using the STRING database. PPIs with interaction scores > 0.4 were used to build a PPI network. Fifty-two genes in brown module, 18genes in red module and 112 genes in yellow module were identified as candidate hub genes by setting the degree value >20 (Fig. 4A–C) Then, we filtered the genes in key modules under the condition of module membership > 0.8 and gene significance > 0.8, a total of 53 genes in brown module, 27 genes in red module and 21 genes in yellow module were screened out. After cross-matching with candidate hub genes in PPI network, 3 genes (CCND1, SOX9, GLI1) in the brown module, 1 gene (CAV1) in the red module and 4 genes (C3AR1, TLR3, LY86, TREM2) in yellow module were selected as hub genes. Expression levels of 8 hub genes were shown in Figure 4D. CCND1, C3AR1, TLR3, LY86, and RREM2 were up regulated in VSs, GLI1, SOX9, and CAV1 were down-regulated in VSs. All changes are statistically significant (*P* < .05 calculated by the student *t* test).

### 3.5. Validation in external datasets

**Figure 5.**
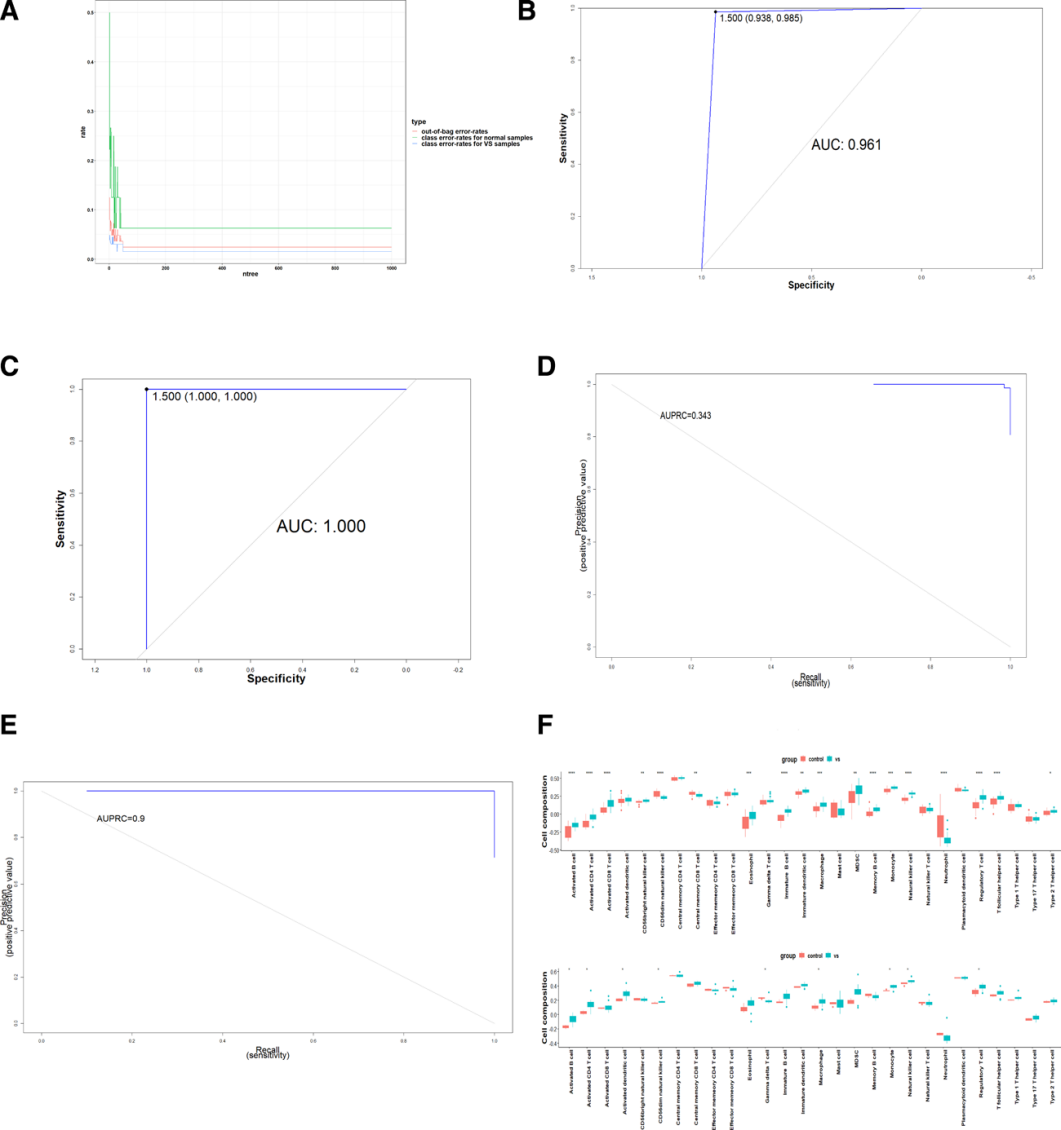
Validation in external datasets. (A) Relationship between the related errors and the number of decision trees in random forests. (B–C) Receiver operating characteristic (ROC) curve of the random forest model in internal cohort and external cohort. (D–E) Precision-recall curve of the random forest model in internal cohort and external cohort. (F) The landscape of immune cell infiltration based on external cohort (bottom) and internal cohort (top) (*P < .05, **P < .01, ***P < .001).

We next used the machine learning random forests algorithm to construct a prediction model based on 8 hub genes. In this study, internal cohort was used to build model and external cohort was used to validated model. After the quantity of decision trees reached 800, the performance of the random forest tended to be optimal (Fig. 5A). Figure 5B–E illustrate the values of AUC and AUPRC. Our model demonstrates robust performance on both the internal (AUC = 0.961, AUPRC = 0.343) and external cohort (AUC = 1, AUPRC = 0.9). Confusion matrix of random forest classifier based on 3 datasets included in our study are shown in Table S1, Supplemental Digital Content, http://links.lww.com/MD/I752. Our random forest classifier was able to distinguish VSs versus normal nerves.

Expression levels of 8 hub genes based on 3 datasets included in our study were shown in Figure S2, Supplemental Digital Content, http://links.lww.com/MD/I753. We also analyzed the immune cell infiltration in external cohort through ssGSEA (Fig. 5F). We found that difference of activated B cell, activated CD4 T cell, immature B cell, Macrophage, MDSC, and Natural killer cell Monocyte remained significant in external cohort. For Eosinophil, Neutrophil, Regulatory T cell, and T follicular helper cell, the same tendency was observed, but the difference was not significant.

### 3.6. Correlation analysis of hub genes and immune infiltration level

**Figure 6.**
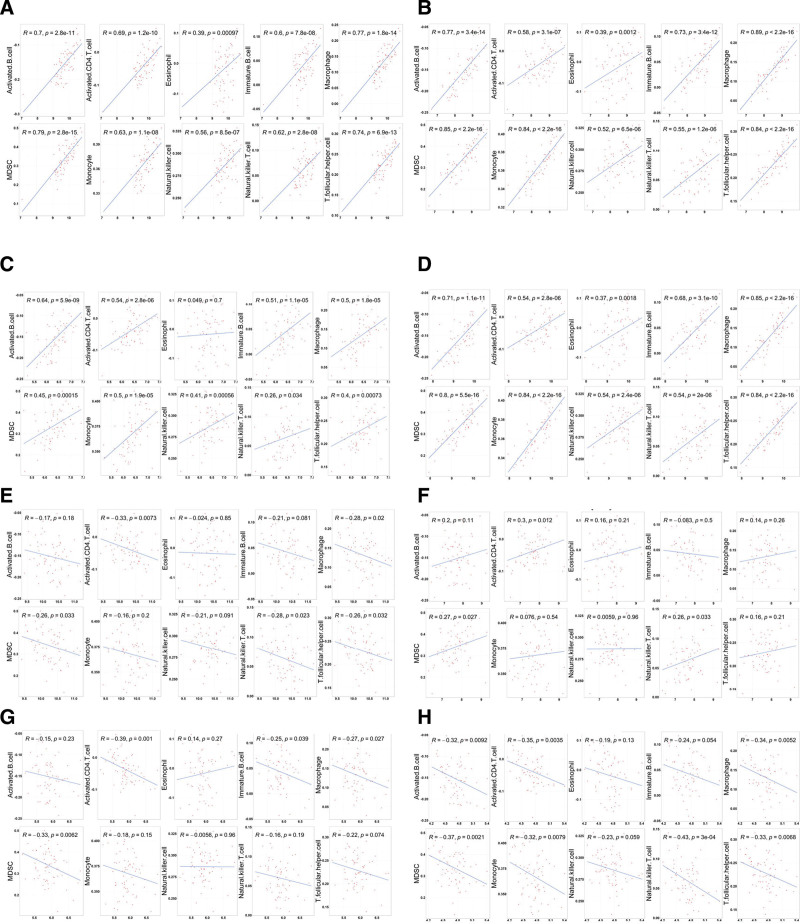
Correlation of 8 hub genes with immune infiltration in VS. (A) C3AR1, (B) LY86, (C)TLR3, (D) TREM2, (E) CCND1, (F) CAV1, (G) SOX9, and (H) GLI1. VS = vestibular schwannoma.

To further understand the relationship between hub genes identified in our study and diverse infiltrating immune cell types, the correlations between hub genes and each immune cell type in VS samples (Fig. 6) were performed by Pearson correlation analysis. Four hub genes (C3AR1, TLR3, LY86, TREM2) in yellow module were positive correlated with the infiltration of immune cells, CCND1, and GLI1 were negative correlated with the infiltration of immune cells.

## 4. Discussion

As the most common tumor in the cerebellopontine angle region, the pathogenesis of vestibular schwannoma is still unclear. In this study, we utilized bioinformatics methods to search for hub genes in vestibular schwannomas and explore their immune infiltration.

First, we applied WGCNA to the gene expression profiles GSE141801 and GSE54934 and identified 13 modules on the top 60% variant genes from 83 samples. Three (red, brown and yellow) out of 13 modules were significantly correlated with VS. Brown and red modules were found to be involved predominantly in collagen-containing extracellular matrix, extracellular matrix organization, cell-cell junction, focal adhesion, cell adhesion molecules. Abnormalities of functions mentioned above may contribute to loss of contact inhibition of growth.^[13]^ Moreover, brown and red modules related to axonogenesis, positive regulation of neurogenesis, signaling receptor activator activity, PI3K-Akt signaling pathway, and EGFR tyrosine kinase inhibitor resistance, which may associate with proliferation of VS.^[14,15]^ We also found that yellow module was related with immune response. Infiltration of immune cells has been extensively reported in various tumors, but few studies have been conducted to evaluate immune infiltration in VSs.

Therefore, we next analyzed immune infiltration in VSs and normal nerves through ssGSEA and found that 17 kinds of immune cells in VSs were identified to be significantly different from those in normal nerves, which provided new insights into the infiltrating patterns of immunocytes in VSs. Correlation analysis showed that natural killer (NK) cells, and regulatory T (Treg) cells were most associated with VS. NK cells have the capacity to directly kill tumor cells, they can also enhance antitumor activity of other immune cells.^[16,17]^ There is mounting evidence that NK cells infiltrate various tumors.^[18]^ Numerous investigations have demonstrated the influence of Tregs in development and progression in a variety of tumors, and Tregs are generally believed to be a significant contributor to tumor immune escape.^[19]^ A widely accepted hypothesis is that infiltration of Tregs with immunosuppressive properties enable the malignant cells to evade the host immune response.^[20]^

Our main results also revealed CCND1, CAV1, GLI1, SOX9, LY86, TLR3, TREM2, and C3AR1 as hub genes for VS. CCND1 encode Cyclin D1, which promotes cell cycle progression during the G1-S phase. CCND1 might be a pivotal downstream target gene in EGFR-driven tumorigenesis and is up regulated in a variety of tumors,^[21,22]^ including VSs.^[23]^ CAV1 is a membrane protein that forms the coat of plasma membrane caveolae.^[24]^ CAV1 may play an inhibitory role in some tumors, and the expression of CAV1 has been proved to be related with better prognosis in some tumor types.^[25–27]^ Stromal CAV1 under-expression has also been associated with autophagy, hypoxia, and oxidative stress.^[28,29]^ GLI1 encodes a member of the Kruppel family of zinc finger proteins. Abnormal activation of GLI1 is the crucial process of Hedgehog/GLI signaling, which has multiple etiological roles in the initiation and progression of a variety of tumors by regulating their critical oncogenic traits such as cell proliferation, metastasis, and cancer stem-like cells fate.^[30]^ SOX9 is a member of the SOX family of transcription factors. it is generally accepted that SOX9 promotes the formation of tumors by regulating cell cycle progression and cell proliferation.^[31]^ However, there are also opposite results showing that SOX9 functioned as a tumor suppressor.^[32,33]^ LY86 encodes protein lymphocyte antigen 86, which also known as MD-1, is a secreted glycoprotein. MD-1 is expressed in various cells, including macrophages, dendritic cells, B cells and monocytes.^[34]^ MD-1 can interact with radioprotective 105 and mediates the response to lipopolysaccharide and regulation of the innate immune system and inflammation on the surface of immune cells, including B cells, macrophages, and dendritic cells.^[35]^ LY86 was identified as a biomarker for the prediction of osteosarcoma, prostate cancer and renal cell carcinoma.^[36–38]^ TLR3 encode toll-like receptor 3, which is a type of pathogen recognition receptor. TLR3 was found to be expressed in immune cells, such as myeloid dendritic cells and intestinal epithelial cells but not in monocytes, polymorphonuclear leukocytes, T, B and NK cells.^[39]^ On the other hand, TLR3 was also shown to be expressed in neurons, astrocytes, and microglia.^[40]^ TLR3 demonstrated a dual role in tumor biology, since studies have associated TLR3 levels with either good or poor clinical outcome in different cancer types.^[41]^ TREM2 is a transmembrane receptor of the immunoglobulin superfamily and a crucial signaling hub for multiple pathological pathways that mediate immunity, which is expressed on myeloid lineage cells such as dendritic cells and resident tissue macrophages. Pan-cancer study indicated that there were positive correlations between TREM2 expression and both stromal and immune cell content in tumor microenvironment.^[42]^ C3AR1 is an orphan G protein-coupled receptor for C3a, which is decomposed by C3. C3AR1 can be expressed by glia, neurons, and infiltrating immune cells in the central nervous system.^[43]^ Recent studies have revealed that a high expression of C3AR1 is positively correlated with a poor prognosis and increased tumor immune infiltration.^[44]^ Four hub genes (C3AR1, TLR3, LY86, TREM2) in yellow module showed moderate positive correlations with the infiltration of immune cells, which may suggest that these 4 genes were upregulated on a variety of tumor-infiltrating immune cells.

Based on the random forest, a prediction model of 8 genes was constructed to distinguish VSs from normal tissues. The results showed that the random forest model of the 8 genes can distinguish VSs and normal tissues both in internal cohort and external cohort. Moreover, we also analyzed the immune cell infiltration landscape of VS and normal control nerves in external cohort through ssGSEA and the results are similar to the outcomes in internal cohort. All of these suggest the results of this study were robust.

Finally, we also analyzed the correlation between 8 hub genes and immune infiltration. We found that 4 hub genes in yellow module were positive correlated with immune infiltration, which suggested these 4 hub genes may contribute to immune cell infiltration. Notably, CCND1, which plays a significant role in cell cycle, was negative correlated with the infiltration of immune cells. it seems that immune infiltration involved in the negative regulation of cell proliferation in VSs; however, this could not be confirmed because the growth rate of VSs were not measured in all samples included in this study.

The present study also has limitations. The patients included in our study lack clinical information such as growth rate of VSs, we cannot determine the exact mechanism of the identified hub genes and immune infiltration in VS.

## 5. Conclusions

In this study, we performed WGCNA in independent gene expression datasets and identified 8 hub genes and some functional biological pathways involved in VS. Furthermore, we have revealed immune infiltration in vestibular schwannomas, suggesting that immune infiltration may play an important role in neural benign tumors of the nervous system. In summary, the present study provides noteworthy directions for future research.

## Author contributions

**Data curation:** Yanpeng Fu.

**Formal analysis:** Yanpeng Fu.

**Funding acquisition:** Yuehui Liu.

**Investigation:** Yanpeng Fu.

**Methodology:** Yanpeng Fu.

**Software:** Yanpeng Fu.

**Supervision:** Yaqiong Zhu.

**Validation:** Liqing Guo.

## Supplementary Material
