# Supplementary material for: Identification of key genes and immune infiltration based on weighted gene co-expression network analysis in vestibular schwannoma
Source: Medicine (Baltimore). 2022 Apr 7;102(14):e33470. doi: 10.1097/MD.0000000000033470 (PMC10082262; doi:10.1097/MD.0000000000033470)
Supplement: Supplementary file 2 [file medi-102-e33470-s002.pdf]

1.1 **Table S1** Demographic information for each of the datasets

	GSE141801		GSE54934		GSE108524	
	Turmor (N=36)	Normal (N=7)	Tumor (N=31)	Normal (N=9)	Tumor (N=10)	Normal (N=4)
Gender:						
Male	23	NA	14	NA	8	2
Female	13		17		2	2
Age(mean±SD)	45.13±12.50	NA	44.5±14.3	NA	42.7±10.28	43.25±17.72
