# Supplementary material for: Identification of key genes and immune infiltration based on weighted gene co-expression network analysis in vestibular schwannoma
Source: Medicine (Baltimore). 2022 Apr 7;102(14):e33470. doi: 10.1097/MD.0000000000033470 (PMC10082262; doi:10.1097/MD.0000000000033470)

Figure S2: Expression level of eight hub genes in three datasets. P values calculated by the student t test.

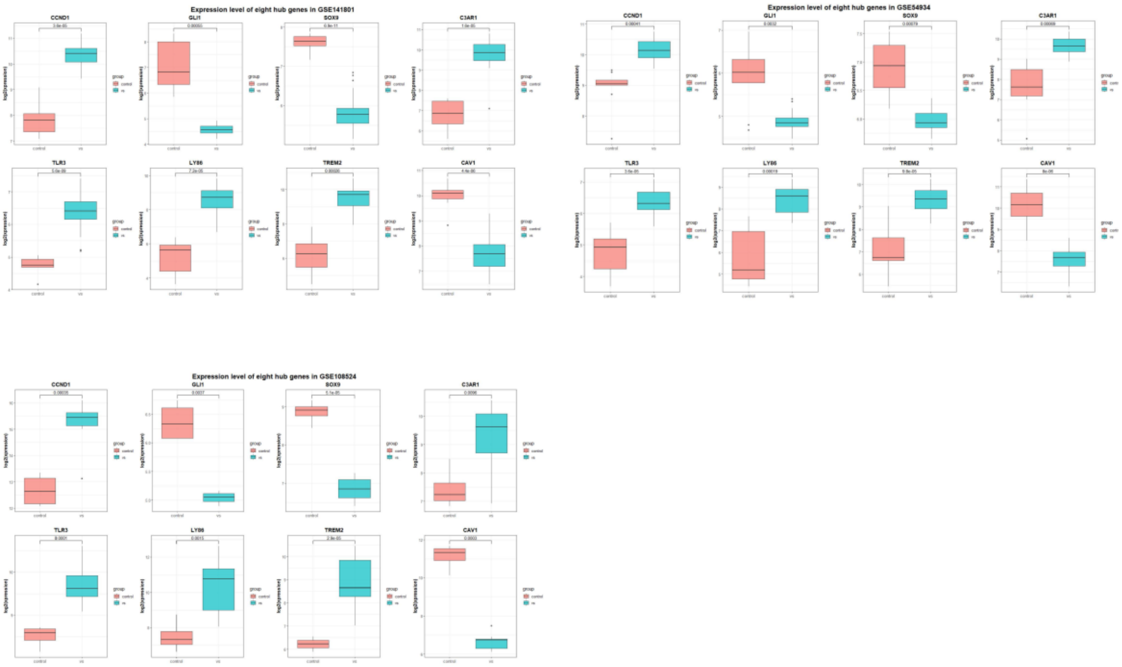

Supplement: Supplementary file 3 [file medi-102-e33470-s003.pdf]
